# Interference of Monoclonal Gammopathy with Fibrinogen Assay Producing Spurious Dysfibrinogenemia

**DOI:** 10.1055/s-0039-1683969

**Published:** 2019-03-19

**Authors:** Francesca Martini, Nadia Cecconi, Aldo Paolicchi, Sara Galimberti, Giulia Cervetti, Gabriele Buda, Mario Petrini

**Affiliations:** 1 Department of Clinical and Experimental Medicine, U.O. Hematology, University of Pisa, Pisa, Italy

**Keywords:** coagulation factors, inhibitor, fibrinogen, fibrin

## Abstract

Abnormal coagulation properties indicative of a dysfibrinogenemia were found in the plasma of an asymptomatic 65-year-old male. An immunoglobulin k light chain was found to interfere with Fg functional assay and coagulation tests (activated partial thromboplastin time, prothrombin time, and thrombin time). Steroid therapy reduced the inhibitory effect (after dexamethasone treatment coagulation test and functional Fg value normalized). Spurious dysfibrinogenemia associated with light chain monoclonal gammopathy of undetermined significance was diagnosed.

## Introduction

Disorders of fibrinogen (Fg) are usually the result of genetic mutations that hesitate in reduced levels of protein (hypofibrinogenemia) or in an abnormal molecule (dysfibrinogenemia). 1 2 3 However, plasma factors or microenvironment can determine an acquired defect of Fg. 4 5 Our goal is to identify the cause of coagulopathy in a 65-year-old man, diagnosed because of a discrepancy between immunological Fg and functional Fg.

## Case Report

An apparently healthy 65-year-old man without a tendency to bleed was referred to our center because of abnormal coagulation assay results ( Table 1 ) that were detected prior to surgery for thyroid nodule. He had never received any anticoagulants. Hereditary deficit was excluded, due to normal coagulation assays tested a year ago. Patient never showed thrombotic or hemorrhagic diseases. Thyroid nodule was removed without any bleeding problems during or after the procedure. At present, the patient is healthy and asymptomatic.

**Table 1:** Patient's coagulation assays

FII	131%	NV 70–130%
FV	103.9%	NV 65–130%
FVII	120.2%	NV 65–140%
FX	97.9%	NV 70–120%
FVIII	102.2%	NV 60–150%
FvW Ag	116%	NV 50–150%
FvW cof. ristocetinico	85.9%	NV 50–150%
FIX	99.0%	NV 60–130%
FXI	93.6%	NV 65–120%
FXII	101.6%	NV 70–130%
Aggr. PLT ADP 2 μM	100%	NV 52–100%
Aggr. PLT ADP 10 μM	100%	NV 64–100%
Aggr. PLT arachidonic ac. 1 mM	100%	NV 70–100%
Aggr. PLT collagen 2 μg/mL	100%	NV 58–100%
Aggr. PLT ristocetin 1.5 mg/mL	100%	NV 79–100%
Aggr. PLT epinephrine 5 μM	100%	NV 64–100%
TT	Invaluable	
aPTT	Invaluable	NV 25,1–36, 5 s
PT	Invaluable	NV 9, 4–12, 5 s
Fibrinogen (Clauss)	Invaluable	
Immunologic fibrinogen	373 mg/dL	

Abbreviations: Ag, antigen; aPTT, activated partial thromboplastin time; FvW, von Willebrand factor; PLT, platelet; PT, prothrombin time; TT, thrombin time.

## Coagulation Assays

Routine coagulation screening revealed unmeasurable activated partial thromboplastin time (aPTT), prothrombin time (PT), and thrombin time (TT). The results of the coagulation tests are shown in Table 1 . No antiphospholipid antibodies were present. Platelet count was normal. Coagulation factor activity assays were performed: activity of factors II, V, X, VIII, IX, XI, and XII was normal. Von Willebrand antigen (FvW Ag) and activity (FvW ristocetin cofactor assay) values were normal. Platelets aggregation assay was normal. PT and aPTT mixing test with normal plasma (1:1) resulted in PT and aPTT correction.

A discrepancy between immunological Fg and functional Fg was revealed: an acquired dysfibrinogenemia was suspected. 6 7

## Inhibitor Detection and Characterization

**Fig. 1:**
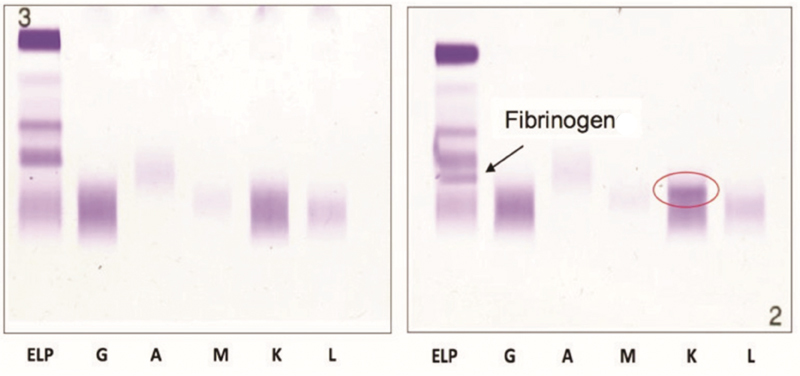
Protein serum immunofixation (on the left) and plasma protein immunofixation (on the right). The circle underlines k monoclonal light chain.

The patient was screened for monoclonal gammopathy. The band of monoclonal light chain k was identified near the band of Fg on plasma immunofixation ( Fig. 1 ).

No clonality was identified on urine and serum immunofixation, possibly because k chains were bound to Fg molecules, absent in serum assay. Bone marrow plasma cells (PCs) were normal (4–5%) but 80% of these PCs were monoclonal on flow cytometric assay (CD138+, CD38+, CD19−, 93.6/5.9% k/lambda intracytoplasmatic ratio).

Treatment with dexamethasone (40 mg/day for 4 days) resulted in an almost complete correction of functional Fg value, and normalization of aPTT, PT, and TT values. After steroid treatment, free light chain (FLC) k values rose as if k light chains were released from Fg molecules. Three months after steroid therapy, aPTT, PT, and TT became abnormal again (prolonged aPTT and PT, unmeasurable TT), along with unmeasurable functional Fg values.

To better understand the inhibitory effect observed on patient's functional Fg, we performed three mixed assays.

- We mixed patient's plasma with normal-health donor plasma (pooled normal plasma [PNP]) in a 1:1 ratio: measured functional Fg in the mix sample was inferior to the expected value; the expected value was determined as weighted average between functional Fg measured in PNP (318 mg/dL) and functional Fg measured in patient's plasma (0 mg/dL unmeasurable). No differences between tests run at 4 or at 37°C were observed, so we could rule out an interaction between the k light chain and glucose part of the Fg molecule. No differences of measured functional Fg after 1 or 2 hours of incubation time were observed. A mixed test made after steroid therapy revealed measured functional Fg in the mix sample comparable with the expected value: steroid therapy reduced the inhibitory effect of patient's plasma (probably related to monoclonal k light chains) on functional Fg.
- We mixed Fg (purified from PNP in a buffer solution) with patient's plasma in different ratios (1:5, 1:2, and 5:1). The measured functional Fg in the three mix samples was inferior to the expected values; the expected value was determined as weighted average between functional Fg measured in PNP (mL PNP × 292 mg/dL) and functional Fg measured in patient's plasma (mL patient plasma × 0 mg/dL unmeasurable). Patient's plasma could inhibit Fg in normal plasma pool as well.
- We mixed purified patient's Fg (buffer solution) with purified Fg from PNP (buffer solution): by increasing the patient's Fg proportion in relation to the normal one, we observed an initial decline in the measured functional Fg value of the mix sample until the ratio of 5:1 was reached. No further functional Fg decline was observed, increasing patient's Fg proportion: the measured functional Fg value of the mix sample reached a plateau as if saturation of the light chains' interfering capacity was achieved.

We screened 20 patients with multiple myeloma (control group) for similarities with this patient's coagulopathy: in four patients we found TT elongation but normal functional Fg values. Plasma immunofixation performed in these four myeloma patients did not reveal a light chain band that run at the same level of Fg molecules. A mixing test with purified Fg from the multiple myeloma patients and Fg purified from PNP did not show any decrease in measured functional Fg values of the mix sample.

## Discussion

Interactions of paraprotein with fibrinopeptide release, fibrin polymerization, or factor XIIIa-mediated cross-linking have already been described. Paraprotein may impair fibrin formation by either antigen–antibody interactions, nonspecific interactions, or increasing plasma viscosity caused by high protein concentration in the blood. This case is particularly unusual because the inhibitory effect on Fg molecules was induced by a single light chain rather than by a complete antibody molecule. There have been described only two previous reports of a light chain inhibitory effect on Fg. 8 9

A specific test to assay the interaction between monoclonal light chain (obtained from patient's plasma) and Fg from PNP will be necessary to surely prove coagulopathy related to monoclonal FLCs. Kotlín et al described a case of myeloma paraprotein immunoglobulin G interfering with fibrin polymerization: enzyme-linked immunosorbent assay (ELISA) and Western blotting experiments confirmed the antibody–antigen interaction of paraprotein with Fg. 10 The normalization of coagulation assays occurred after melphalan–prednisone treatment as in our patient after dexamethasone treatment. In our patient we could exclude an antibody–antigen interaction because a single light chain is involved.

No alterations in platelet aggregation test were observed, so an interaction at the Fg's binding site for platelets IIb/IIIa glycoprotein was excluded.

As no hemorrhagic events were reported by the patient, an interaction at the Fg's binding site for coagulation factor XIII (fXIII) or a fibrinopeptide A (FpA) impaired release was unlikely.

We suspect an interference with fibrinopeptide B release or fibrin polymerization.

Three months after steroid therapy, aPTT and PT were abnormal but measurable, while TT and functional Fg were unmeasurable. Moreover, PT and aPTT mixing test with normal plasma (1:1) resulted in PT and aPTT correction, while functional Fg was still unmeasurable (mixing assay 1). Therefore, the PT and aPTT tests are less sensitive than the TT test and functional Fg assay. Considering the above, the inhibitory agent present in the patient plasma may be low titer, even if we did not measure it directly. We could only measure FLC k values, which were inversely related to the inhibitory effect on functional Fg (after steroid treatment FLC k values rose, while functional Fg increased).

This case is interesting because studying coagulation assays allowed us to diagnose FLC-MGUS (monoclonal gammopathy of undetermined significance), which needs to be followed up.

## References

[1] Mosesson M W The roles of fibrinogen and fibrin in hemostasis and thrombosis Semin Hematol 1992 29 03 177 188 1641664

[2] Casini A Blondon M Tintillier V Mutational epidemiology of congenital fibrinogen disorders Thromb Haemost 2018 118 11 1867 1874 30332696 10.1055/s-0038-1673685

[3] Casini A Blondon M Lebreton A Natural history of patients with congenital dysfibrinogenemia Blood 2015 125 03 553 561 25320241 10.1182/blood-2014-06-582866 PMC4296015

[4] Mammen E F Coagulation defects in liver disease Med Clin North Am 1994 78 03 545 554 8170258 10.1016/s0025-7125(16)30146-8

[5] Post G R James L Alapat D Guillory V Cottler-Fox M Nakagawa M A case of acquired dysfibrinogenemia in multiple myeloma treated with therapeutic plasma exchange Transfus Apheresis Sci 2013 48 01 35 38 10.1016/j.transci.2012.06.021 22842111

[6] National Committee for Clinical Laboratory Standards.NCCLS Document H30-A: Procedure for the Determination of Fibrinogen in Plasma; Approved Guideline Villanova, PA National Committee for Clinical Laboratory Standards 1994

[7] Palareti G Maccaferri M Manotti C Fibrinogen assays: a collaborative study of six different methods Clin Chem 1991 37 05 714 719 2032326

[8] Dear A Brennan S O Sheat M J Faed J M George P M Acquired dysfibrinogenemia caused by monoclonal production of immunoglobulin λ light chain Haematologica 2007 92 11 e111 e117 18024387 10.3324/haematol.11837

[9] Llobet D Borrell M Vila L Vallvé C Felices R Fontcuberta J An acquired inhibitor that produced a delay of fibrinopeptide B release in an asymptomatic patient Haematologica 2007 92 02 e17 e19 17405747 10.3324/haematol.10575

[10] Kotlín R Sobotková A Riedel T Acquired dysfibrinogenemia secondary to multiple myeloma Acta Haematol 2008 120 02 75 81 18841003 10.1159/000160182

